# Opioid death projections with AI-based forecasts using social media language

**DOI:** 10.1038/s41746-023-00776-0

**Published:** 2023-03-08

**Authors:** Matthew Matero, Salvatore Giorgi, Brenda Curtis, Lyle H. Ungar, H. Andrew Schwartz

**Affiliations:** 1 Department of Computer Science, Stony Brook University, Stony Brook, NY USA; 2 Department of Computer and Information Science, University of Pennsylvania, Philadelphia, PA USA; 3 National Institute on Drug Abuse, National Institutes of Health, Baltimore, MD USA; 4 Department of Psychology, University of Pennsylvania, Philadelphia, PA USA

**Keywords:** Computer science, Computational science, Social sciences

## Abstract

Targeting of location-specific aid for the U.S. opioid epidemic is difficult due to our inability to accurately predict changes in opioid mortality across heterogeneous communities. AI-based language analyses, having recently shown promise in cross-sectional (between-community) well-being assessments, may offer a way to more accurately longitudinally predict community-level overdose mortality. Here, we develop and evaluate, TrOP (*Tr*ansformer for *O*piod *P*rediction), a model for community-specific trend projection that uses community-specific social media language along with past opioid-related mortality data to predict future changes in opioid-related deaths. TOP builds on recent advances in sequence modeling, namely transformer networks, to use changes in yearly language on Twitter and past mortality to project the following year’s mortality rates by county. Trained over five years and evaluated over the next two years TrOP demonstrated state-of-the-art accuracy in predicting future county-specific opioid trends. A model built using linear auto-regression and traditional socioeconomic data gave 7% error (MAPE) or within 2.93 deaths per 100,000 people on average; our proposed architecture was able to forecast yearly death rates with less than half that error: 3% MAPE and within 1.15 per 100,000 people.

## Introduction

The United States has been attempting to tackle an opioid epidemic for over two decades, with age-adjusted opioid-related deaths increasing by 350% over 20 years from 1999 to 2020^1^. One of the key challenges to the epidemic is that its underlying driving force seemingly changes across time and communities, for example, from prescription drug abuse to cheap and readily available synthetic opioids (e.g. fentanyl)^2^. There is suspected to be large heterogeneity among risk factors per community across the US^3^. Traditional methods to capture community characteristics, focused on economic, broad healthcare, and survey outcomes, only capture a fraction of what matters during the everyday lives of community members, especially as communities change from year to year^4^. With the epidemic shifting over time it is often difficult to properly allocate resources to areas until it is already too late^5,6^. By using more fine-grained community representations, accounting for more precise differences in communities, it may be possible to better forecast opioid mortality, improve preparation, and ultimately mitigate outbreaks.

Here, we evaluate the use of recent advances in AI-based sequence modeling^7,8^ as well as fine-grained characterization of communities from language^9,10^ in order to predict rates of future annual opioid deaths at the county level across the US. We attempt to demonstrate the feasibility of using language from Twitter with modern AI-based techniques to forecast year-to-year changes in opioid mortality. Robust death estimates are often released more than a full year after the last day of the year^11^. Using modern AI-based community forecasts utilizing social media language can greatly speed up the response.

The need for anticipating large increases or decreases in deaths has not gone unnoticed. Recently, the CDC has implemented the “OverdoseData2Action” (OD2A) plan, a collaboration with state and local governments to track changes in opioid-related use^12^. A key component of this plan is the collection of timely and accurate data to help health officials better understand the issues and prepare responses^12^, including the launch of a center for forecasting and outbreak analysis^13^.

While we believe this to be an early evaluation of a digital language-based opioid forecasting system at scale—capturing counties across most of the US—it joins a recent increase in work to leverage data analytics to better understand substance and opioid use and especially their relationship with socio-demographics^14^. Most related are those studies that leveraged language data from social media, sometimes examining counts of opioid-related words (e.g. fentanyl) and use rates^15^ and increasingly using more sophisticated AI-based or machine learning methods, to predict opioid use and outcome rates^16,17^. Many of these studies are focused on specific regions (e.g. a single US state)^18^ or specific groups of people such as medicare beneficiaries or adolescents^19,20^.

The use of social media data brings important challenges in data veracity. First, Twitter users skew young; they are not a randomly sampled sub-population^21^. Second, there are bots that actively post to Twitter that do not represent a real person and their interactions with their community. Finally, there is concern over whether the persona shown on Twitter authentically matches a person’s true self. However, a developing body of work has demonstrated that with some care for these issues, representative health and well-being statistics can often be predicted from such noisy, but extremely large, data. Previous works have proven capable of predicting useful outcomes using noisy social media data, including predicting alcohol consumption rates^10^, estimating well-being^9^, and a variety of other health statistics and community behaviors^22–25^.

These works, like the present study, avoid the assumption that social media data is unbiased, and rather evaluate estimates derived from it against accepted representative figures. In our case, we are evaluating against predictions of *future* years’ rates using social media data only from prior years. Past works that demonstrated the capacity of social media to produce community characterizations have focused mostly on point estimates of a sample population at a single time span; Past work has evaluated cross-sectional modeling, while here we develop and evaluate a longitudinal model that estimates future changes.

We use the *transformer* sequence model—adopted parallel attention-based sequential modeling with the transformer architecture^7^. Within language and vision, AI-based sequence modeling has recently moved from recurrent neural networks (e.g. RNNs like LSTMs and GRUs)^26,27^ to transformer networks^28^. However, public health studies that are geared towards prediction often opt instead for traditional cross-sectional modeling with linear/logistic regression, support vector machines, or gradient boosting trees^29–31^). This new architecture allows sequences to be analyzed with multiple representations, called attention-heads, which give it a strong ability to see how each step of the sequence interacts with past steps. Transformer models are often first pre-trained on general language modeling tasks^32,33^ and then used as sentence encoders or fine-tuned to a specific task^34,35^. However, even when trained from scratch on a specific task, particularly for forecasting, they can offer strong results^8^.

Our contributions include: (1) proposing *Tr*ansformer for *O*piod *P*rediction (TrOP), a transformer-based sequence modeling technique to predict *future* county opioid death rates leveraging Twitter language representations which we make available via GitHub, (2) demonstrating the feasibility of a multi-regional longitudinal method for scalable state-of-the-art yearly forecasts of opioid deaths, (3) evaluating the unique benefits of the transformer-based model as compared to standard and modern alternatives, and (4) highlighting Twitter county linguistic patterns that reliably increase/decrease in years prior to changes in county opioid death rates.

Outside of cross-sectional studies, social media and online forum data have also been used for time-series modeling such as emotion tracking^36,37^ and early detection of mental health issues^38^. While these works look at the progression of language over time, they are restricted to the psychological states of individual people. Modeling at the community level adds additional complexity as there are three levels of modeling: individual language “utterances" (e.g., status updates), which are aggregated to individual people, which are in turn aggregated into a community (e.g., a county).

## Results

### Overview of TrOP and county sample

We evaluated TrOP against other machine learning and sequence models at predicting future yearly opioid mortality from past yearly opioid-related mortality together with past Twitter-based county representations. As described in detail within the “Methods” section, TrOP uses a multi-headed attention-based transformer network; the other sequence models included linear auto-regressive models and non-linear recurrent neural networks (RNNs).

We used language data derived from the County Tweet Lexical Bank (CTLB)^23^. To ensure adequate sampling, CTLB was restricted to counties that had at least 100 users with 30 or more tweets, overall years of CTLB collection, resulting in 2041 counties. We then found an overlap of these counties with those available from CDC Wonder^39^ that had yearly opioid-related death rates available for all queried years, resulting in our final 357 counties. These 357 counties have a total population of 212 million people, covering 65% of the total population at the time of our last year of data (2017). Our topic vectors are then derived based on *yearly* language from these counties.

### Overall results

We evaluated TrOP utilizing both (1) past opioid mortality and (2) past language use representations at the community level, and compared it to models utilizing recurrent deep learning methods (e.g. RNN) and linear auto-regression, as well as, heuristic *baselines* using the previous year’s estimate (last(1)) and the average of the last 4 years (mean(4)).

The results of our models when trained on both opioid death history and language history are described in Table 1, including heuristic baselines leveraging only past knowledge of opioid-related deaths. Overall, TrOP ’s predictions were more accurate than the comparative models when using 3 years of history (3 years was found to be optimal for all models). Both neural models, utilizing non-linear techniques, achieved 1–2% lower error than the linear model. Our proposed model, TrOP, had the lowest percent error down to 2.92% while using the same history as the other models.

**Table 1:** Comparison of TrOP to alternative models with the inclusion of yearly topic data and using optimal yearly history length per model type.

Model (history)	MAPE	MAE
*Baselines*		
Last (1)	16.16	5.76
Mean (4)	36.61	11.67
*Linear*		
Ridge AR (3)	6.31	2.63
*Deep learning*		
Recurrent Neural Net (3)	3.99	1.64
TrOP (3)	2.92*	1.15*

Model error is reported via mean absolute percent error (MAPE) and mean absolute error (MAE) with MAE representing the number of deaths per 100,000 our predictions are off by on average. All models performed quite well but the transformer-based architecture scored less than half the error of the traditional linear model, likely due to being able to extract multiple representations of the language sequence. Bold results represent best in column and * indicates a significance with *p* < 0.01 using a paired *t*-test with respect to the RNN model.

We evaluated the performance of our models compared to and combined with socio-economic variables (SES) in Table 2. Here, Ridge SES is a linear ridge regression model trained using past knowledge of 7 socio-economic variables; log median household income, median age, percentage over 65, percent female, percent African American, percent high school graduate, and percent bachelor graduate. Due to 7 years of SES history not being available for 20 counties from our primary analyses, the results reported here used a slightly smaller (and thus we also report our model results on the same subset for comparison). Besides use on their own, the 7 SES variables were also integrated into TrOP and the other autoregressive models by concatenating them within the input vectors of history alongside the past language representations and past opioid deaths. Notably, the SES variables on their own were predictive beyond the baselines but in no situations were they able to contribute to any of the language-based autoregressive models toward an improvement.

**Table 2:** Evaluation of TrOP and alternative methods with the inclusion of socio-economic variables.

Model (history)	MAPE	MAE
*Baselines*		
Last (1)	16.22	5.75
*Linear*		
Ridge SES (3)	6.77	2.75
Ridge AR (3)	6.31	2.62
Ridge AR + SES (3)	6.55	2.67
*Deep learning*		
Recurrent Neural Net (3)	3.99	1.62
Recurrent Neural Net + SES (3)	4.18	1.67
TrOP (3)	2.96*	1.16*
TrOP + SES (3)	3.37	1.34

Errors are reported as MAE and MAPE (deaths per 100k). Here, due to not all counties having available socio-economic variables for all years (2011–2017) our dataset is reduced by 20 counties, thus we report the same approaches from Table 1 with predictions limited to available counties. Ridge SES is a ridge regression using only past socio-economic variables, whereas models denoted “+ SES" are using past opioid death rates, language data, and socio-economic variables. Bold results represent best in column and * indicates a significance with *p* < 0.01 using a paired *t*-test with respect to TrOP + SES.

**Fig. 1:**
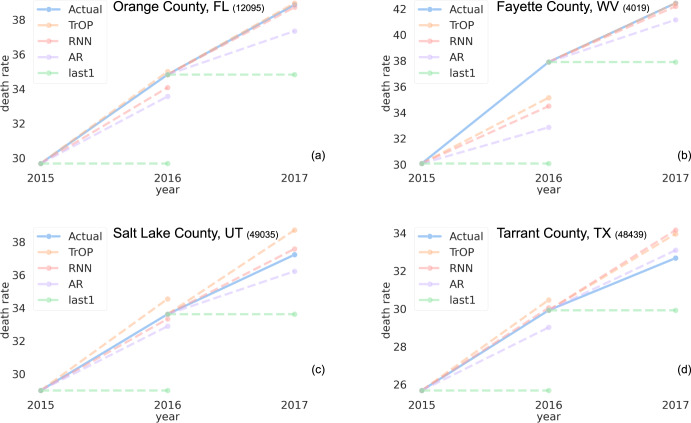
Forecasting model error for selected counties. Visualization of forecast errors, in deaths per 100,000, for all of our machine learning models as well as the last 1 baseline. We highlight 2 counties where TrOP performed best, Orange County, FL (**a**) and Fayette county, WV (**b**), as well as 2 counties where the RNN and linear model are each best, Salt Lake County, UT (**c**) and Tarrant County, TX (**d**), respectively.

To exemplify what our models had learned, both good and bad, we investigated predictions for specific counties from TrOP as well as the other statistical models. Our goal was to examine both counties where TrOP gave accurate forecasts and those where it failed to do so. A select few counties are shown in Fig. 1 with examples showing both low and high errors for TrOP. In general, it appeared that TrOP often overestimated the rate of change when it got the prediction wrong, whereas the other models most often underestimated it. TrOP was also capable of getting an incredibly accurate (near 0 error) forecast for a handful of counties, which even the best counties for the other models did not achieve.

**Fig. 2:**
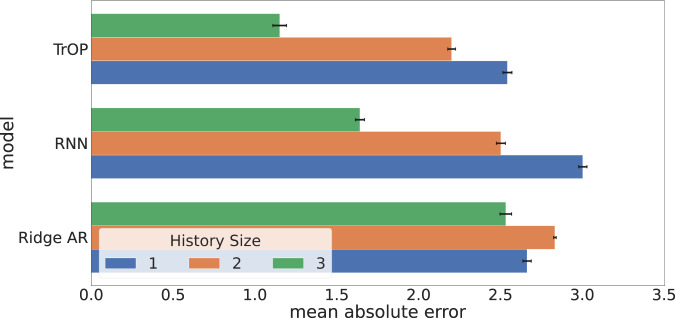
Model error by available history. Examining the trend in mean absolute error (MAE) rates, with 95% confidence intervals, based on available history for multivariate versions of our models. We found that all models perform best with 3 years of data available. For the deep learning approaches, there was a large drop when increasing from 2 to 3 years of history noting the utility in measuring the change in language over time.

Figure 2 describes how our models behaved when trained with <3 years of data. For the linear model, there was not much of a change overall while both non-linear neural networks were better able to utilize relationships between the language data across years. However, while they both saw noticeable decreases in error at 2 or 3 years, they struggled when restricted to only 1 year of data.

**Fig. 3:**
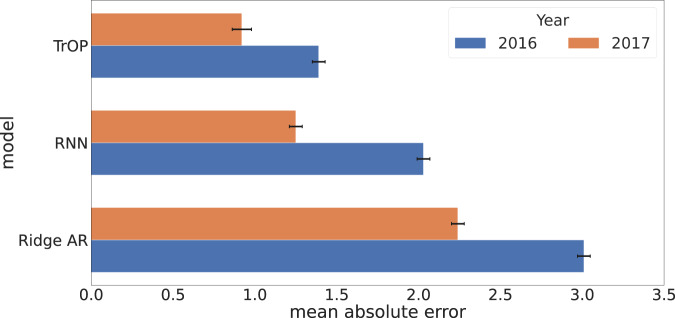
Model error by test year. Mean absolute error (MAE) and 95% confidence intervals across our 2 test years for each statistical model. TrOP showed lower error in both years compared to other approaches. The overall gain fromTrOP appeared to be in having a more robust prediction of 2016, with a reduction of 0.6 MAE when compared to RNN. However, all models followed the same trend of 2016 being a bit harder to predict than 2017.

To better understand how our models predicted year to year, we broke down the mean absolute error of each of our multivariate models by test year, shown in Fig. 3. Overall we found that each model’s error was mostly stable over both years, but did see a clear trend in 2016 being a harder year to forecast.

**Fig. 4:**
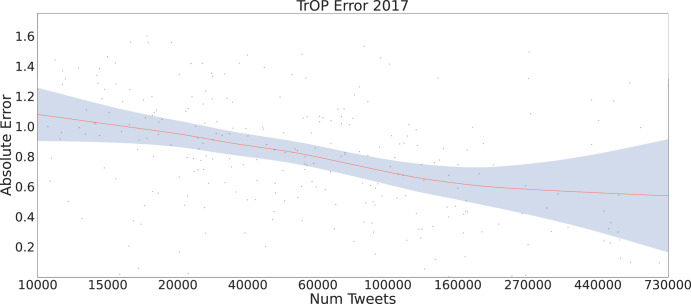
Model error by Tweets per county. Impact of the number of tweets on error, as measured by deaths per 100k, per county for the test year (2017). The line on the graph is fit with a LOWESS regression^64^ with a shaded region indicating the 95% confidence interval. Overall TrOP shows a trend of decreasing error as counties have increasingly more language to build topic representations from.

Lastly, we investigated TrOP ’s error with respect to data availability per county, shown as a LOWESS fit in Fig. 4 with a 95% confidence interval. In general, we saw a decrease in error as the number of tweets increases with the effects tailing off at 160,000 tweets.

### Univariate modeling

One could model the progression of time using only a single variable of interest (e.g., opioid-related deaths) and build a model that operates on this single feature at each time-step.

We investigated the utility of such an approach by comparing our model’s performance using only a univariate input versus a multivariate one; as shown in Table 3. Here, we found that all models saw a considerable drop in predictive power when the language features were removed. These results highlight how each model benefited from the inclusion of language-based features from Twitter. Some models, such as the linear autoregressive ridge saw only a small increase in error, 0.6% MAPE, while both non-linear models saw roughly 5 times that with an increase in the error of roughly 2−2.6% MAPE.

**Table 3:** Comparison of performance across all of our proposed machine learning models in a univariate context and their ideal history length in the number of years they have access to.

Model (history)	MAPE	MAE
*With Language*		
Ridge AR (3)	6.31	2.63
Recurrent Neural Net (3)	3.99	1.64
TrOP (3)	2.92*	1.15*
*Without Language*		
Ridge AR (3)	7.09	2.93
Recurrent Neural Net (3)	6.97	2.84
TrOP (3)	6.93*	2.81*

Errors are reported in both mean absolute error and mean absolute percent error. All models performed slightly worse when trained on just a sequence of opioid death rates(no language features) but the transformer architecture continued to be better than other approaches. Bold results represent the best model per configuration(with or without language) and * indicates a significance with *p* < 0.01 using a paired *t*-test with respect to the Gated Recurrent model of the same configuration.

### Language changes prior to opioid mortality changes

To gain insight into the individual language patterns that reliably predicted future opioid deaths, we evaluated the relationship between changes in each of the topics prior to changes in the outcomes for both 2016 and 2017. We used a linear model with the standardized change in opioid rate as the dependent variable and standardized prior change in the topic as the independent variable, correcting for prior year opioid deaths as an additional covariate. We tested for the significance of the effect between each topic and opioid change, correcting for the false discovery rate with the Benjamini–Hochberg procedure^40^.

**Fig. 5:**
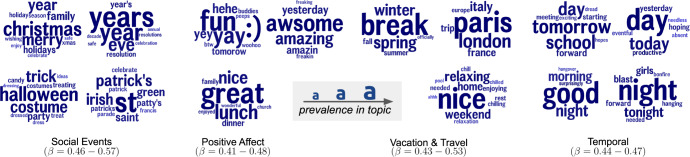
Language associated with lower opioid death rates. Topics longitudinally predictive of lower opioid death rates: yearly change in language (2014–2015, 2015–2016) negatively associated with future year change in opioid death rates. Individual topics (clusters of semantically related words) are represented by their 15 most prevalent words (larger is more prevalent). Association (*β*) is the coefficient from standardized multiple linear models, adjusting for prior change (*p* < 0.05; Benjamini–Hochberg adjusted for false discovery rate).

**Fig. 6:**
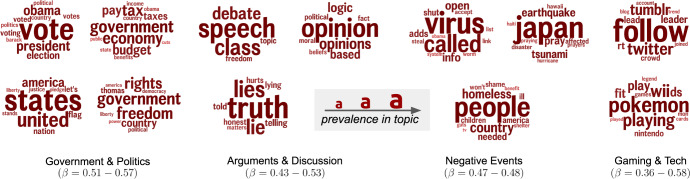
Language associated with higher opioid death rates. Topics longitudinally predictive of higher opioid death rates: yearly change in language (2014–2015, 2015–2016) positively associated with future year change in opioid death rates. Individual topics (clusters of semantically related words) are represented by their 15 most prevalent words (larger is more prevalent). Association (*β*) is the coefficient from standardized multiple linear models, adjusting for prior change (*p* < 0.05; Benjamini–Hochberg adjusted for false discovery rate).

Figures 5 and 6 highlight topics that either decrease or increase prior to increases in opioid death rates for 2016 and 2017. Over 50 topics were significantly associated and are also available in Supplementary Table 4. The displayed clusters were manually grouped by first examining the individual topics that have significance *p* < 0.05 and then manually paired among other topic clouds that showed similarities. Each cluster of individual topics represents semantically similar language associated with opioid deaths.

## Discussion

We evaluated how accurately one can forecast U.S. county opioid deaths using a modern AI-based sequence modeling technique, *transformers*. We found that TrOP, our model utilizing transformers, was able to achieve only 3% mean absolute percentage error (MAPE), reducing the error in half as compared to a non-transformer model with the same input which achieved 7% MAPE. While there has been a large increase in opioid-related deaths across the US recently, the rates of change differ substantially by county^2^. A model that operates on data unique to each county is necessary to handle the heterogenous and evolving nature of the epidemic^3^, rather than models based on national-level general trends.

Traditional data sources fall short of providing annual county-level measurements. For example, one could use socio-demographic representations for each county as covariates, but these features are often fairly static and do not capture the rapidly changing landscape. To this end, we proposed using open-source (e.g. publicly available) language data from social media sites. Public social media offers a fast and ecological window into the community and regional trends of the opioid epidemic. The language features that our method relies on are easy to collect and, now that open-source software is available, fast to process into an aggregated representation. Our model’s capabilities are strengthened by modern AI-based language processing techniques at the level of prediction rather than running each individual tweet through a contextual embedding modeling^41,42^ which would take considerable computing power.

We compared the transformer-based TrOP to multiple machine learning-based alternatives spanning regularized linear models and recurrent neural networks. We found that both with and without language (multivariate and univariate), the transformer offered the most predictive power and had an average error of only 1.15 deaths per 100,000 when language data was included. The key distinction between transformers and the other models is the multi-headed attention mechanism which enables multiple composites of past states^7^. The RNNs we used had the standard neural-network attention, so we suspect the multi-headed attention mechanism was the basis of the superior performance. This highlights the capabilities of transformers for time-series in the domain of natural language processing, which past works have not clearly demonstrated^37^. For a full comparison of the number of attention heads used in our transformer-based model, see Supplementary Table 1.

While language data helped all models, it did not reduce errors in predictions for the linear model as much as the neural network-based methods. This is likely due to the aggregated language data being more informative when modeled in a non-linear fashion, which is often the case in language-based AI systems, as the interactions between changes in yearly language are complex.

Past work has found social media language can partially capture covariance between socio-economic variables and drinking^10^ or well-being^9^, cross-sectionally. However, in a longitudinal design as in the current study, it is less clear whether socio-economic (SES) variables are beneficial and if so whether social media will out-predict them. Thus, we evaluated the longitudinal predictive value of socio-economic variables used both by themselves as well as integrated into our models. As shown in Table 2, we first show that the SES variables do in fact predict beyond the prior-year baseline. However, once SES variables were added to our models they did not appear to provide additional benefits. This suggests the covariance accounted for by SES is already covered within social media-based features. This corroborates previous cross-sectional research suggesting social media-based predictions seem to capture county socio-economics^10,43,44^.

While our proposed model TrOP performed best overall in terms of prediction errors, there were still cases where it is unable to outperform alternative approaches in predicting future trends, as described in Fig. 1. These case studies may be useful in indicating what behaviors these counties have in common and what is driving the opioid epidemic in their region.

Further, as shown in Fig. 2, the deep learning models not only benefited from the inclusion of language features but also older language. We believe this is due to the superior sequential modeling powers of both the RNN and Transformer networks and their ability to attend to specific time-steps using their respective attention-mechanisms. Both neural models picking up on signals in older language points towards changes in language as a key indicator of opioid abuse. This highlights the need for larger data in the temporal dimension for training big neural networks. We also explored dynamic window sizes (e.g. training on data of multiple lengths between 1 and 3) and show the results in Supplementary Table 2.

As the opioid epidemic is always changing, it is important to analyze how our model(s) were able to forecast different years’ changes in the death rate. Figure 3 shows that all models had a similar ability to predict 2017 a bit easier than 2016. This is likely caused due to the death rates across years having a large increase from 2015 to 2016, the largest in all years we’ve collected, which explains the model’s inability to forecast as accurately. With counties in the east seeing the largest jump in death rates from 2015 to 2016 and the Midwest seeing the largest jump from 2016 to 2017^45^. However, all of the models still offered somewhat accurate predictions given this shift in the evaluation data which suggests that forecasting with language can help combat distributional shifts in the temporal dimension. Investigating error further, we show the impact of the number of tweets per county on TrOP ’s absolute error in Fig. 4. The trend, plotted as a LOWESS fit, shows counties have more language data, in raw tweets, show less error on average than those with less. This is likely due to the topic representations being more robust when there is more language from per county to aggregate.

While we present our work with a focus on prediction accuracy, there is also insight from the language trends themselves. Presented as clusters of topics we show language that has moderate negative correlations (0.40 ≤ *r* ≤.57) and positive correlations (0.36 ≤ *r* ≤0.58), in Figs. 5 and 6, respectively. The positively correlated language can be thought of as a potential risk factor for increases in opioid abuse and negative language can be viewed as a lower risk.

Notably, for negative correlations, we found lots of discussions focused around positive events, particularly family and social events (e.g. holidays) and personal engagement (e.g. work/school and travel). This use of positive language implies a sense of “anti-despair" and optimism. Alternatively, for positively correlated topics, we see discussions that are less involved with one’s personal social circle and instead focused on more worldly events (e.g. politics, negative events). While these topic clouds show a sense of empathy (e.g. homelessness and veterans of wars) it is towards events or ideas that lean more negatively in thought.

Additionally, we include Supplementary Figs. 1 and 2, which are the groupings of topics significantly correlated with our *model’s* predicted forecasts, rather than observed changes, allowing us to gain insight into what the model may be weighing towards each prediction.

In this work, we set out with two major goals in mind: (1) to build a tool that can be used to forecast future trends in the United State’s opioid epidemic and (2) to examine community-level language behaviors and trends that may be useful in gaining insight as to what might be driving these changes within individual counties. Our first goal is achieved by TrOP which achieves a mean absolute percent error of 2.92%. We highlighted certain counties where our model underperforms alternative methods, showed predictive power based on available years of history, broke down error rates based on the year of prediction, and also examined the relationship between the amount of language data and forecasting error. Finally, we then used our county-level topic vectors to gain insight into the language itself, where we found various changes in yearly topics that correlate both positively and negatively with changes in opioid death rates.

TrOP demonstrated the ability for county-level forecasting not otherwise available, but there are limitations to its application in its current form. Firstly, TrOP is trained only on data from 2011 to 2015 and evaluated over 2016–2017, only two years prospective to the training years. This restriction for the current study was due to (1) the timing of outcome data release and (2) the collection and availability of county-level time-specific curated tweets as part of the county tweet lexical bank (CTLB)^23^ while simultaneously implementing the novel use of transformers. While TrOP may aid with the former issue, the latter issue could be resolved as such methods are more available and with more projects focused on geospatial social media language curation.

Secondly, the predictive power of TrOP will be impacted by distributional shifts in death rates from year to year that cannot be explained via language features shifts alone. While the evaluation years 2016 and 2017 contained larger shifts than most demonstrating some robustness to such time-series “shocks”, this could be further improved with the development of more models that incorporate more temporally dependent or fine-grained structured socio-economic variables than those currently available at the county-year level.

## Methods

### Formulation and design

We sought to forecast the amount of opioid-related deaths per US county each year. To do this we employed various techniques from both time-series modeling and deep machine learning. We formulated our goal in both the univariate case (Eq. (1)) and multivariate (Eq. (2)), where some function *f* (e.g. our learned model) uses information from previous time-steps to predict the next time-step, with error *ϵ*. In the multivariate case of Eq. (2), each time-step contains a *D*-dimensional feature vector, here *D* = 20, concatenated (indicated as ;) with the observed univariate values from Eq. (1) totaling 21 dimensions per time-step. These additional 20 dimensions represent yearly language use per US county aggregated from Twitter.

1 $$f({Y}_{t},{Y}_{t-1},...,{Y}_{t-n})+\epsilon ={Y}_{t+1}$$

2 $$f(Y_{t};{X}_{t}^{d},Y_{t-1};{X}_{t-1}^{d},...,Y_{t-n};{X}_{t-n}^{d})+\epsilon ={Y}_{t+1}$$

Due to most counties across America having an increasing amount of opioid-related deaths per year in our dataset, we applied a single integration step where a difference is taken between neighboring time-steps to alleviate the issue of non-stationarity in each time-series. This also means that the model is designed to forecast the *change* in opioid-related deaths per county. While a single difference does not guarantee stationarity, each subsequent difference reduces the amount of history length by 1. Thus, we framed the problem to rely only on 1 integration step due to only having 7 years of total history across both train and test. After predicting the change, to get the final prediction a reconstruction step must occur between the last observed value and the predicted value, shown in Eq. (3) where *Y*_*t*_ is the observed value and *P*_*t*_ is the predicted change.

3 $${Y}_{t}+{P}_{t}={Y}_{t+1}$$

### Dataset and preprocessing

To collect the yearly opioid death numbers we queried the CDC Wonder tool for the cause of death. We included the following multiple causes of death codes for counting towards opioid deaths: opium, T40.0; heroin, T40.1; natural and semi-synthetic opioids T40.2; methadone, T40.3; synthetic opioids, T40.4; or other and unspecified narcotics, T40.6. The output from a CDC Wonder query is a comma-separated value (CSV) file that contains the county code, total population, crude death rate, and age-adjusted death rate for each county per year. We queried CDC Wonder for the years 2011–2017 and filtered to counties that had reported over all of these years.

However, for some counties, the age-adjusted rate was suppressed when collected from CDC Wonder. To accommodate for this we queried CDC wonder again, this time to get the age breakdown of the population per county and then constructed yearly age terciles. We then used these terciles to bucket the population count per county and fit a linear regression model to the crude death rate. The residuals from this fit were used to generate our age-adjusted death rate, shown in Eq. (4). This was done for each year of our dataset, using that year’s age and opioid data. Here, *O*_crude_ and *P*_residual_ are the crude opioid death rate and residual unique to the county, respectively, and *O*_mean_ is the average crude opioid death rate across all counties within a given year. These generated age-adjusted rates are treated as our labels for training our machine learning models.

4 $${O}_{{\rm {age}}\_{\rm {adj}}}={O}_{{\rm {crude}}}-{P}_{{\rm {residual}}}+{O}_{{\rm {mean}}}$$

Next, to collect covariates for our time-series we started with language data previously curated from Twitter. The dataset we used was a subset of the County Tweet Lexical Bank (CTLB)^23^, which contains aggregated data across US counties. CTLB is comprised of both bag-of-words style features (uni-grams, word count) and bag-of-topics^46^. While pre-aggregated Latent Dirichlet Allocation (LDA) topic vectors existed, they were not broken down by year so we instead used the uni-grams and word count data to generate our own yearly topic vectors. To ensure quality representations CTLB used the following inclusion criteria per county: (1) a county needs 100 active users and (2) each user must have a minimum of 30 tweets. However, this is a requirement over the entire temporal span of CTLB (2011–2016), thus for topic vectors spanning a single year, a county may not have guaranteed 100 active users with 30+ tweets in any given year.

Therefore, in the case that a county did not meet the requirements for a given year their language data were replaced with the average of the topic vectors from other counties in that year. This occurred for a small number of counties each year and counties were selected only if they had at most 2 years missing. All counties were represented by a 2000-dimension LDA topic vector for each year and our final included counties were limited by those that had reported their opioid death rates for all collected years (2011–2017) to the CDC, resulting in a final 357 counties.

Due to the dataset size (*N* = 357) we performed a dimensionality reduction across our 2000 dimension topic vectors. We chose to apply a non-negative matrix factorization (NMF)^47^ due to showing good performance in past works that relied on dimensionality reduction techniques on language^34,48^. We learned the NMF reduction over the collection of training years (2011–2015) and for each year’s 2000 dimension topic vector the NMF reducer was then applied to bring each county’s language data to 20 dimensions.

Lastly, we explored a version of the dataset using socio-economic variables from US Census data. Here, we pulled 7 socio-economic variables over the years 2011–2017 for all counties in our dataset that had this information available. This resulted in a somewhat smaller dataset than our Twitter-only version, dropping 20 counties overall, showing the benefit of using publicly available language data over traditional sources. These 7 variables include: log median household income, median age, percent age over 65, percent female, percent African American, percent high school graduate, and percent bachelor graduate.

### Model design

We explored a variety of models that have been shown to work in the time-series domain such as linear autoregressors^49–51^ as well as deep learning sequential models that have shown promise in temporal natural language processing (NLP)^36,37^. Additionally, transformer^7^-based models were considered, which have shown state-of-the-art performance in many NLP-based tasks^32^.

### Transformers

The transformer network has seen great success since its inception in 2017 and popularization by the BERT language model in 2018^32^. These types of models handle sequences much differently than recurrent neural networks, notably transformers do not recur down the sequence but rather process each step in parallel. This is achieved by generating a positional encoding that gets added to the inputs of the network as well as utilizing residual connections so that this positional information is not forgotten at higher levels in the network. Additionally, transformers use multi-headed attention giving the network multiple internal representations of how sequential steps may relate to one another. While there is evidence that attention heads can be pruned for inference^52^, models are typically trained in a multi-headed fashion. An overview of attention and the multi-head combination are shown in Eqs. (5) and (6).

5 $${\rm {hea{d}}}_{i}={\rm {Attn}}({\rm {Query,Key,Value}})={\rm {softmax}}\left(\frac{Q{K}^{{\rm {T}}}}{\sqrt{{d}_{k}}}\right)V$$

6 $${\rm {MultiHead}}({\rm {Query,Key,Value}})={\rm {concat}}({\rm {hea{d}}}_{1},{\rm {hea{d}}}_{2},...,{\rm {hea{d}}}_{h}){W}^{{\rm {O}}}$$

Here, the attention is determined by the scaled dot-product of query, key, and value vectors where the query maps to a key–value pair. For example, the query may be time-step 1 with values at time-steps 1–3. The key is what determines how related the pairings of query and values are. Since we run this attention across the input sequence itself, this is called “self-attention”, as opposed to traditional attention networks which often calculate attention across outputs of an encoder to build a better representative input into a decoder-based model to perform prediction or generation. The scaling factor is $\sqrt{{d}_{k}}$ which is the square root of the number of dimensions in the key vector. After calculating the attention for the query, keys, and values across *h* attention-heads (in our model *h* = 3), each of their representations is concatenated and one last linear transformation is applied using weight matrix *W*^O^.

Oftentimes, when transformer models are used for NLP tasks a model pre-trained as a language model is used to generate contextual embeddings, or the language model is directly “fine-tuned” to the task of interest^33^. However, language models have yet to produce embeddings for hierarchical representations beyond the sentence level, thus preventing us from using an off-the-shelf model for county-level language and applying it to our forecasting task. Instead, we built a small transformer network from scratch that operates on pre-aggregated feature representations rather than individual words or sentences to use for our task of yearly change in opioid-related deaths.

Due to the small size of our dataset, we implemented a single (1) layer transformer network rather than the common 6 or more^8,53^. Transformers usually have tens to hundreds of millions of parameters so that they can encode a lot of information that would transfer to other tasks (e.g. language models)^54,55^. We believed that even though transformers typically thrive in large data scenarios, it was possible to train a robust model that is adequately downsized to match the data available. Our model used the following configuration: 1 transformer layer, fixed sinusoidal positional embeddings, 3 self-attention heads, input dimensions of 21, feed-forward hidden size of 128, and drop out of 0.20.

For the final prediction from our model, we took the representation for the last year, *H*_*t*_, and used that as input into a linear layer which then forecasted the change for each county. We trained all models in PyTorch and PyTorch Lightning^56,57^, using AdamW^58^ for optimization, and Optuna^59^ for hyperparameter tuning over a held-out development set. The following parameters and search spaces were explored: Learning Rate (5e−3–5e−5), Weight Decay (0–1.0), Dropout (0.1–0.5 with steps of 0.05), and Hidden Size (1–16 with steps of 2 for univariate, and 32–256 with steps of 16 for multivariate).

Lastly, we explored running the transformer network in a bidirectional format analogous to a recurrent network^60^. We found that this neither improved nor degraded the predictive capability of our model. We believe that this may be due to having such a short sequence where the multi-headed attention can already extract the relevant interactions.

**Fig. 7:**
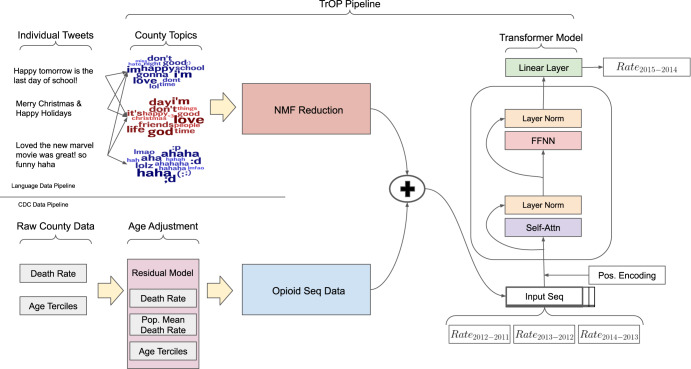
Data pipeline and architecture of the TrOP system. Flow of TrOP from data collection to prediction. We pull from 2 data sources, the CDC for opioid and age-related information and Twitter for language data for each county. We then build the feature representations for each data source and concatenate them for each year (indicated by ’+’). Finally, the yearly sequence data is passed to our transformer predictive model to forecast the rate of change in opioid death rate per county.

The final pipeline for our data processing and model are shown in Fig. 7, which covers data intake to final prediction. Before loading data into our models, the time-series has a single integration step applied to it (neighbor differencing) so that our model is trained to predict change in rates rather than the raw rate. The example in the figure is the configuration when training with a history of 3, such that the inputs are the changes in opioid death rates for [2012–2011], [2013–2012], and [2014–2013] with the final prediction being [2015–2014]. In the case of our 2 test years, the sequence would slide down one step for each prediction (e.g. [2013–2012], [2014–2013], [2015–2014] predicting [2016–2015]).

### Recurrent networks

Gating-mechanism-based recurrent neural networks such as gated recurrent units (GRU)^27^ and long–short-term memory (LSTM)^26^ cells were also considered as potential models. Historically, recurrent neural networks have shown great promise for sequence and time-series modeling^61^ with GRU-based networks offering similar or better performance than LSTM on smaller data^62^ while being a less complex model. Thus, our main focus is on leveraging GRU cells, but we show results based on LSTM architecture as well in Supplementary Table 3.

These types of recurrent networks are different than transformers as they have to move down each step of the sequence one-by-one, using 2 types of inputs. The first input is the features representing the current time-step and the second input is the previous hidden state from the previous time-step(s) (i.e. the recurrent step). The final representation that is used to decode the hidden representations is a weighted sum of the hidden states across the time-steps as defined by a neural network attention mechanism^63^. Additionally, our RNN networks used a bidirectional representation, where the forward and backward networks have their hidden states concatenated at each time-step, which was found to improve predictive power.

### Linear models

Based on the history of linear modeling in the time-series domain, we opted to use an L2 regularized (Ridge) linear regression as one of our baseline models. These linear models are simple and quick to implement while still often giving near state-of-the-art results. We trained our ridge regression using a gradient descent approach, with the same frameworks as our deep learning models.

### Heuristic baselines

In addition to the linear and RNN models, we also considered two simple baselines that have proven useful for time-series models^37^: (1) predicting the last observed value again (no change) and (2) predicting the mean of all *k* observations.

All procedures were approved by the University of Pennsylvania Institutional Review Board #6 under exempt status.

### Reporting summary

Further information on research design is available in the Nature Research Reporting Summary linked to this article.

## Supplementary information

REPORTING SUMMARY

Supplementary Information

## Data Availability

County language data is available through the 2011–2017 County Tweet Lexical Bank^23^. Our yearly aggregated language topic vectors along with historic opioid-related deaths, per county, will be added to the repository: https://github.com/wwbp/county_tweet_lexical_bank.

## Code availability

The model definition, data loader, and train/test loop for TrOP will be available at the following repository: https://github.com/MatthewMatero/TrOP.
