# Author Correction: Opioid death projections with AI-based forecasts using social media language

**DOI:** 10.1038/s41746-023-00793-z

**Published:** 2023-03-17

**Authors:** Matthew Matero, Salvatore Giorgi, Brenda Curtis, Lyle H. Ungar, H. Andrew Schwartz

**Affiliations:** 1 Department of Computer Science, Stony Brook University, Stony Brook, NY USA; 2 Department of Computer and Information Science, University of Pennsylvania, Philadelphia, PA USA; 3 National Institute on Drug Abuse, National Institutes of Health, Baltimore, MD USA; 4 Department of Psychology, University of Pennsylvania, Philadelphia, PA USA

**Keywords:** Computer science, Computational science, Social sciences

Correction to: *npj Digital Medicine* 10.1038/s41746-023-00776-0, published online 08 March 2023

The Acknowledgements statement is now amended to: ‘Support for this research was provided by the following grants: (1) Large-scale Data Scientific Assessment of Unhealthy Alcohol Consumption Among Front-Line Restaurant Workers (NIH-NIAAA R01 AA028032) and (2) Advancing Language-based Analyses of Social Media to Reliably Monitor Variation in Population Mental Health (NIH-NIMH R01 MH125702). Additionally, this research was in part supported by the Intramural Research Program of the NIH (ZIA DA000628).’ The original Article has been corrected.

