# Supplementary material for: Opioid death projections with AI-based forecasts using social media language
Source: NPJ Digit Med. 2023 Mar 8;6:35. doi: 10.1038/s41746-023-00776-0 (PMC9992514; doi:10.1038/s41746-023-00776-0)
Supplement: Supplementary file 2 — Supplementary Information [file 41746_2023_776_MOESM2_ESM.pdf]

Supplementary Information

Supplementary Figures

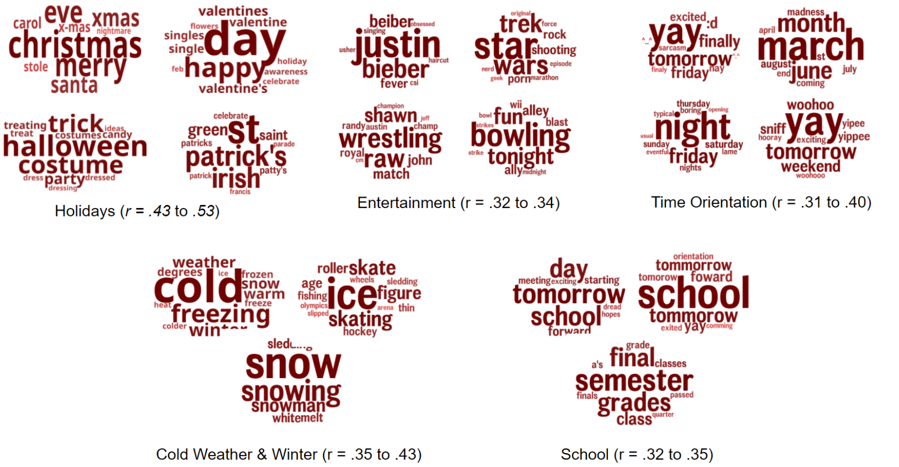

Supplementary Figure 1 Clusters of topics that were found to be positively correlated with the forecast from our best performing model (TROP). Individual topics were (manually) clustered together with others discussing similar ideas and labeled with their cluster category and Pearson correlation range ($p < .05$). The larger the word within an individual cluster, the more often it appears in that topic.

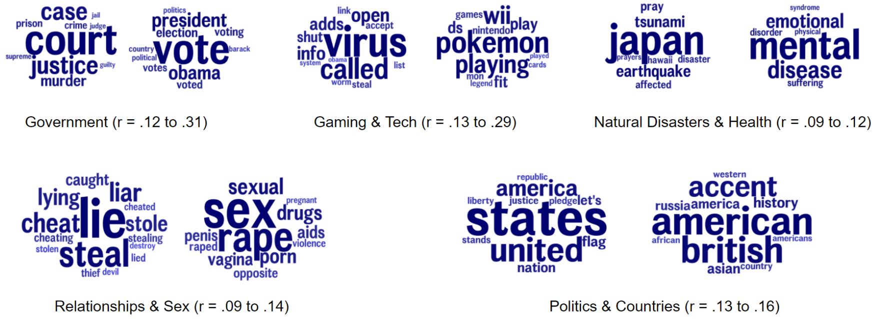

Supplementary Figure 2 Clusters of topics that were found to be negatively correlated with the forecast from our best performing model (TROP). Individual topics were (manually) clustered together with others discussing similar ideas and labeled with their cluster category and pearson correlation range ($p < .05$). The larger the word within an individual cluster, the more often it appears in that topic.

Supplementary Tables

Model (history)	MAPE	MAE
TROP $_{1head}(3)$	5.44	2.18
TROP $_{3head}(3)$	**2.92***	**1.15***
TROP $_{7head}(3)$	4.08	1.58

Supplementary Table 1 Impact of number of attention heads on TrOP predictive power. Number of heads is restricted such that the number of feature dimensions (21) is divisible by the number of heads (1,3,7).

Model (history)	MAPE	MAE
TROP (1+)	4.13	1.60
TROP (2+)	5.39	2.17
TROP (3)	**2.92***	**1.15***

Supplementary Table 2 Evaluation of using dynamic window size vs fixed (e.g. training only sequence of length 3 vs 1-3 or 2-3). TROP is always evaluated using the longest sequence it saw in training (3). We found that training a fixed length of 3 performs better than other configurations for TROP .

Model (history)	MAPE	MAE
With Language		
LSTM (3)	5.53	2.28
GRU (3)	3.99	1.64
Without Language		
LSTM (3)	6.81	2.77
GRU (3)	6.97	2.81

Supplementary Table 3 Comparison of popular RNN cell types; LSTM and GRU. We found that GRU to be better than LSTM when language variables are considered but LSTM had lower error when trained on a univariate sequence of just past death rates.

	Topics associated with decreased mortality (negative coefficients)			Topics associated with increased mortality (positive coefficients)		
rank	id	*β*: 95%CI	most prevalent terms	id	*β*: 95%CI	most prevalent terms
1	918	[-0.62, -0.51]	christmas merry year family holidays holiday xmas season wishing celebrate enjoy safe jesus eve x-mas feliz blessed spirit gifts celebrating	1360	[0.52, 0.63]	virus called open info adds shut accept steal link list worm system obama spreading trojan confirmed clinton scandal wall barack
2	639	[-0.61, -0.51]	christmas merry eve xmas santa carol x-mas stole nightmare	878	[0.52, 0.62]	vote president obama election voting voted votes country political politics barack government congress campaign bush america vice bill poll debate
3	911	[-0.6, -0.49]	christmas presents shopping gifts xmas present gift wrapping merry wrapped wrap eve cards tree bought opening opened x-mas santa buying	486	[0.51, 0.61]	that's thing guess there's isn't mine volcano donald meets tornado ash bothering roast blinded routine europe messing practically
4	117	[-0.6, -0.49]	christmas tree lights putting decorations xmas decorating spirit holiday decorate decorated presents trees holidays carol x-mas tradition lighting elf underneath	914	[0.49, 0.6]	government freedom rights country thomas political democracy liberty america society power nation human state civil american states war justice united
5	1747	[-0.59, -0.48]	happy year halloween holidays safe	709	[0.47, 0.58]	opinion opinions logic based political fact moral beliefs philosophy argument logical facts society simply FALSE values statement agree ideas behavior
6	49	[-0.58, -0.47]	paris london italy france trip europe germany rome england travel leaving tour uk spain ireland canada french booked york australia	1645	[0.47, 0.58]	american british accent history asian america russia country western african americans native culture china russian ancient european roman europe indian
7	962	[-0.58, -0.47]	day happy valentines valentine's valentine single singles awareness holiday celebrate flowers feb couples lovers romantic february chocolates celebrating sweetest sweetheart	1866	[0.46, 0.58]	speech class debate topic freedom presentation interesting discussion giving prepared speaking prepare debating preparing kings oral public excellent speaker today's
8	1978	[-0.58, -0.46]	st patrick's irish green saint patty's patricks celebrate patrick parade francis celebrating feast ireland pinch whiskey celebrated nicholas snakes festivities	1464	[0.46, 0.57]	states united america flag nation let's justice stands pledge liberty republic longer offending americans allegiance fear grew indivisible country american
9	439	[-0.57, -0.46]	shopping christmas grocery clothes xmas online shoppin spree lunch mall minute shop store shoe centre cart x-mas walmart stores deals	387	[0.45, 0.56]	government economy tax budget pay taxes country income benefits obama state public cuts debt health jobs federal education poor billion
10	1772	[-0.57, -0.46]	holiday season holidays chance poison enjoy favorite weeks bank enjoying spoil cloudy booked public hoped inn hangover oak cheer remedy	45	[0.43, 0.54]	bill ted excellent kill gates kennedy bailey murray clinton adventure gate electric science dollar electricity passing talks scrubs microsoft topped
11	367	[-0.56, -0.44]	halloween costume trick party treating candy costumes treat dressed dress ideas dressing parties outfit scary october witch decorations scare pirate	107	[0.42, 0.54]	july happy fireworks fourth safe display bbq holiday independence celebrate america firework parade celebration blow bonfire celebrating setting lighting cookout
12	1496	[-0.54, -0.42]	break spring winter summer fall officially semester formal hurry quarter seasons autumn begins olympics midterms offically lasted snows	1107	[0.42, 0.54]	people country homeless needed america ill children shame won't shelter guts benefit tv eating haiti meds elderly treatment hundreds thousands
13	1942	[-0.54, -0.42]	santa christmas dear claus naughty monica barbara cruz secret xmas presents brought elf jolly maria dressed suit ana lap workshop	118	[0.41, 0.53]	pokemon playing wii play ds fit nintendo games mon legend cards played trade sports version ash original shiny playin master
14	453	[-0.53, -0.41]	great lunch nice dinner family enjoyed church wonderful afternoon sunday kids evening shopping meeting hubby yesterday lovely relaxing visit spent	1099	[0.41, 0.53]	country freedom men served veterans remember lives serve america serving women died american military forces sacrifice soldiers protect service bless
15	768	[-0.53, -0.41]	dress prom shopping wedding dresses wear bought fancy shoes formal homecoming outfit rehearsal clothes dressed picked fitting graduation wearing heels	800	[0.41, 0.53]	court justice case murder prison crime supreme judge jail guilty trial lawyer criminal protest innocent legal cases witness law ruled
16	278	[-0.53, -0.41]	week days work school weeks starting till yay holiday holidays half wednesday schedule stressful hectic yikes yippee craziness untill yipee	283	[0.41, 0.53]	japan earthquake tsunami pray affected disaster hawaii prayers hurricane praying haiti japanese natural earthquakes victims flood tokyo pacific damage chile
17	1578	[-0.53, -0.41]	night tonight girls blast forward hanging needed bonfire babysitting sleepover girly partying favorites bestie girlies	1133	[0.38, 0.5]	point fact longer including remember country america veteran amount states check wrote blank united honor thankful support freedom soldier troops
18	748	[-0.52, -0.4]	day today yesterday hoping productive eventful absent needless	1412	[0.37, 0.5]	men women woman women's ladies young grown stare international mice equal rarely attractive male female men's boyz woman's manly prefer
19	1803	[-0.52, -0.4]	night sleep tonight rest hoping night's needed decent sleeps stink	1089	[0.37, 0.5]	man woman women men successful wise wife tells treat deserves impress independent pregnant woman's naked forest satisfy loses protect treats
20	18	[-0.52, -0.4]	thankful family happy thanksgiving friends wonderful blessed safe enjoy holiday grateful blessings wishing turkey holidays healthy families health greatful extended	865	[0.36, 0.49]	truth lies lie telling told lying honest hurts matters compared tells lied hide liar tiny honesty secrets hidden liars believes
21	237	[-0.52, -0.4]	tomorrow day today work hoping forward it'll dreading technically	523	[0.36, 0.49]	ne ut sh nt pr st ve ss fr ch ll pe ar ing ab ther rt ck ad tt
22	1546	[-0.52, -0.4]	good night morning surprisingly hangover facebookers	806	[0.36, 0.49]	en som har med nu om tt den kan min lite fr vi du ra bra gon ver ut upp
23	1028	[-0.52, -0.4]	day long work tomorrow ready bed exhausted stressful relax hectic stretch meetings bedtime resting	1320	[0.36, 0.48]	er har til en med som der den om du min kan vi 8d meg nu bare rt han tak
24	945	[-0.52, -0.4]	years year eve year's resolution resolutions safe decade celebration annual tradition hallows previous provided encourage gladly promising nicer	686	[0.36, 0.48]	az meg rt van ny bb ki es jo mint tt ra ut ban sem mar este fog aj vid
25	722	[-0.52, -0.39]	happy thanksgiving turkey thankful feast holiday stuffing stuffed holidays parade oven canadian ham coma dinners traditional tradition pies indians	913	[0.36, 0.48]	bit :/ ly http rt gay lesbian join national clicking ow donate states ally gd youth recognize sending artists legends
26	1394	[-0.51, -0.39]	night saturday friday tomorrow spending sunday forward spend sat thursday exciting takers partying babysitting plans babysit planned	448	[0.35, 0.48]	ga di yg ada yang ini lagi mau apa sama ke saya dan aku nya tau hari dah anak tp
27	1485	[-0.51, -0.39]	day today good longest dragging	1709	[0.35, 0.48]	ich die ist der das es du bin den mein von hat mal um gut dem tag german wer des
28	1436	[-0.51, -0.39]	bed sleep tired night early tonight goodnight goin exhausted calling hrs zzzzz worn yawn retire	892	[0.35, 0.48]	lie steal cheat liar lying stole caught cheating stealing thief lied cheated stolen devil destroy liars someones corner depressed crime
29	63	[-0.51, -0.39]	tomorrow school day forward starting meeting exciting hopes dread returning yikes grin chin	371	[0.35, 0.47]	fact facts useless evidence presence proof random accept absence notice completely proven reading checking existence realize simply prove actual skipped
30	66	[-0.5, -0.38]	cold freezing winter weather snow warm degrees frozen freeze colder heat ice wind warmer toes heater wet chilly froze bloody	585	[0.34, 0.47]	law order illegal laws legal arizona citizen business allowed states marathon physics required banned legally criminal government ban gravity property
31	712	[-0.5, -0.38]	early bed morning tomorrow work bright wake heading bedtime release rise awake goodnight earl healthy morn wayy rested ridiculously	1178	[0.34, 0.46]	xa wave er :\ sem vi fr ver pr tt upp inn vu
32	70	[-0.5, -0.38]	good day pretty feels twas hung chilled	1524	[0.34, 0.46]	people rude selfish stupid ignorant assholes arrogant they're pathetic racist act minded jerks idiots immature asshole dealing bunch greedy comments
33	1616	[-0.5, -0.38]	day today good hay nice hitting hittin ruined stack straw	1624	[0.33, 0.46]	:/ ms http video youtube fm rt posted dc thomas kevin commented uploaded tumblr shares 8d demo orleans preview unsure
34	1220	[-0.5, -0.38]	awsome amazing yesterday amazin freakin freaking absolutly comming finaly chilled jade	1983	[0.32, 0.45]	india indian jai proud country indians ram delhi anna independence nation pakistan gandhi republic salute mumbai common mata shame baba
35	505	[-0.5, -0.38]	early morning woke sleep bed night late mornin awake ugh wake yawn caught sleepin sleepy nap midnight couldnt morn earl	1837	[0.31, 0.44]	ja iv nyt ss pit ty ole ku tt vain vi kin mist eli ist ep
36	1250	[-0.5, -0.38]	day long gonna looong loooong boring ahead todays tomorrows tomorrow's tiring	1244	[0.31, 0.44]	return tax owe taxes favor money returns pay file income claim paid office returned sales property cost government debt bucks
37	196	[-0.5, -0.37]	yay tomorrow finally friday :d excited nay sarcasm ^_^ finaly ^.^	971	[0.31, 0.44]	missed rip miss patrick loved sad forgotten passed died greatly dearly remembered beloved uncle goodbye neil mj sadly harris truely
38	1666	[-0.5, -0.37]	saturday weekend sunday friday monday party excited forward thursday coming busy plans sat evening planned afternoon weekends bbq possibly flags	1750	[0.3, 0.44]	he's guy he'll thinks doesn't brother boyfriend he'd poor tells hero hasn't adorable sees jerk dad's sweetheart handsome talks convinced
39	813	[-0.5, -0.37]	haha lol :p :d	380	[0.3, 0.43]	american idol watching ellen crystal lee casey pie voted idiot vote native psycho auditions simon ai eagle audition billy african
40	437	[-0.5, -0.37]	week weekend great forward hope fast monday end coming start hoping ready sunday flew	71	[0.29, 0.42]	situation personal issues issue statement problem deal current concern multiple considered previous situations problems dealing involved avoid general manner information
41	174	[-0.5, -0.37]	warm cold blanket nice fuzzy inside freezing weather cozy blankets heating cuddle wrapped brings winter snuggle snuggie peeing electric pad	1074	[0.29, 0.42]	thunder rain storm lightning hail storms lightening wind lighting struck thunderstorm raining strike clouds thunderstorms strikes tropical heavy hurricane winds
42	1032	[-0.5, -0.37]	day great today yesterday hope nice ^.^	1051	[0.29, 0.42]	y'all doin yeah hey todd how's swear livin hows drake i'ma sheesh demon thang askin
43	36	[-0.5, -0.37]	good day morning hope great begining optimistic	1929	[0.29, 0.42]	pay save starting money dear lets continue hear mr country lives freeze cut cutting benefits risk president increase risks agree
44	1499	[-0.49, -0.37]	morning woke early bed breakfast coffee waking 8: late saturday woken dressed o'clock sickness noon jog pt sunrise downstairs workout	997	[0.28, 0.42]	police officer arrested cops officers killed line streets safe cop security law department report duty station robbed development jail crime
45	1640	[-0.49, -0.37]	night good mornin al sweetie	1454	[0.28, 0.42]	real fake act pretend pretending aren't tan genuine plastic bitches acting honest smiles lying claim hiding arent mature boobs mask
46	1602	[-0.49, -0.37]	day long work tomorrow atleast whew relaxing tomorow longest favorites continuing	623	[0.28, 0.41]	people lives peoples thier people's heads opinions wonders ruin honest pathetic minds involved else's selfish statuses syndrome amazed houses jealous
47	1228	[-0.49, -0.37]	nice relaxing weekend home enjoying rest needed chilling chill relaxation chilled pool ahhh relaxed relax catching hubby resting ahhhh deserved	443	[0.28, 0.41]	matthew chase christopher gave dave jesse lindsay lives brandon news reed king stanley allen tate sheldon justin zachary brett lohan
48	1189	[-0.49, -0.37]	morning tomorrow sunday saturday afternoon early leaving church kenny o'clock waking picking rogers foward volunteering	830	[0.28, 0.41]	follow twitter tumblr leader rt lead crowd account joined blog trend advice updates leaders instructions shortly suit topic capacity updating
49	1409	[-0.49, -0.37]	night fun tonight girls great party interesting eventful girlies crew bonfire sleepover hella karaoke twas	1510	[0.27, 0.41]	evil sin anger pride wrath greed hatred lust sins destroy pure deadly lies envy proverbs enemies wicked enemy destruction innocent
50	726	[-0.49, -0.37]	good day today hope feels invisible dash surprisingly impossible sees mia	872	[0.27, 0.41]	black white yellow swan racist belt pitch color asian pokemon brown grey keys dressed gray colored sheep racism colors shades

Supplementary Table 4 Linguistic patterns that were associated with change in observed prospective opioid death rate. Specifically, associations between annual topic changes associated with next years annual opioid mortality change resulting from a multiple linear regression, controlling for prior year opioid mortality rate. All associations were significant at $p < .05$ adjusting for false discovery rate.

Supplementary Discussion

Error and Sensitivity Analysis For the main study, the neural network hyper-parameters were chosen based on subsetting training data with a development set. To better explain performance of TROP we performed a few ablations regarding architecture choices. Specifically, we varied the number of attention heads used and the application of a dynamic training size for years of history. Supplementary Table 1 shows the performance of TROP using 1, 3, and 7 attention heads. While both configurations with multiple heads perform better than single head attention, the model using 7 heads appears to be over-tuned compared to 3 heads.

We also tested the performance of TROP when trained using a non-fixed window size as reported in Supplementary Table 2. Instead of training on instances that were always the same length (e.g. 1,2 or 3), the model was trained over sequences of dynamic length (e.g. 1 or more), which increases the number of training instances at the expense of observing the full history size for each instance. Evaluation was still done utilizing maximum window size, matching the application setting. We found that training with dynamic window size did not yield a performance benefit over simply training the model exclusively on the longest possible sequences. This suggests the change in language over time is a stronger signal when more years are considered, and thus adding in shorter sequences does not improve predictive power. We thus utilized the simpler approach, fixed windows with max history for the main TROP results.

Comparison of RNN Cell Types We show the performance of LSTM style RNNs in Supplementary Table 3. We found that LSTM performs a little better on univariate sequences but considerably worse when using the multivariate language setup. We believe this is due to GRUs being better at learning representations over small datasets.

Explainability of TROP through Language While we present our work with a focus on prediction accuracy, there is also insight from the language trends themselves. Presented as clusters of topics we show language that has moderate positive correlations ($.31 \leq r \leq .53$) and low to moderate negative correlations ($.08 \leq r \leq .31$) with TROP 's predicted output, in Supplementary Figure 1 and Supplementary Figure 2 respectively. The positively correlated language can be thought of as potential risk factors for increases in opioid abuse and negative language can be viewed as lower risk. Notably, for positively correlated topics, we see lots of discussions around various holidays, entertainment such as music, movies, or sports, and school. We believe this language use is indicative of white suburban areas. Alternatively, for negatively correlated topics, we found much different language use with more discussions centered around government and politics as well as natural disasters and mental health. We believe the increased discussion of politics within these topics are coming from more educated areas and that communities which are more open about their mental and emotional health show a lower risk of opioid abuse.

Language and Opioid Use Supplementary Table 4 presents the complete list of fifty topics found to be most highly associated with with change in opioid death rates.
